# Exploring the Effects of a School Mindfulness-Based Intervention in French Primary Public Schools—A Pilot Study

**DOI:** 10.3390/pediatric17020031

**Published:** 2025-03-05

**Authors:** Jessica Monsillion, Lucia Romo, Rafika Zebdi

**Affiliations:** 1 UR 4430 Clipsyd, Department of Psychology, Université Paris Nanterre, 200, Avenue de la République, 92001 Nanterre Cedex, France; 2 Hôpital Raymond-Poincaré, 104 Bd Raymond Poincaré, 92380 Garches, France

**Keywords:** school mindfulness-based interventions, school, children, anxiety, depression, school well-being

## Abstract

**Background/Objectives**: School-Mindfulness-Based Interventions (SMBIs) have shown positive effects on children’s mental health by enhancing emotional regulation and present-moment awareness. This pilot study explored the effects of an SMBI program on anxiety, depression, mindfulness, emotional awareness, executive functioning, and school well-being among French primary school students (mean age = 116 months; SD = 9.06). **Methods**: A quasi-experimental longitudinal design was employed, with assessments at pre-intervention (T1), post-intervention (T2), and follow-up (T3). Data were analyzed using repeated-measures ANOVA. **Results**: The study found no statistically significant changes across all assessed dimensions. The lack of significant results may reflect factors such as the program’s duration, small sample size, reliance on self-report measures, and contextual challenges like the COVID-19 pandemic. However, slight numerical trends in anxiety and mindfulness scores suggest potential benefits that larger studies may better detect. **Conclusions**: While no significant improvements were observed, the findings highlight important considerations for SMBI implementation in schools in France and internationally. Future research should address current limitations by increasing sample sizes, employing multi-informant assessments, and integrating SMBIs with complementary approaches, such as social-emotional learning. Extending the program duration or incorporating booster sessions may enhance effectiveness. Embedding SMBIs into school curricula through a whole-school approach could foster the sustainable and impactful integration of mindfulness practices into daily school life.

## 1. Introduction

From school hours to learning methods, school systems in the contemporary Western world are shaped by the unique laws and operational frameworks of each country. This study explores the effects of a School-Mindfulness-Based Intervention (SMBI) in France, addressing a research gap while providing insights for adapting SMBIs in French-speaking countries with similar educational systems, focusing on shared global goals like emotional regulation, attention, and prosocial behaviors. The French public educational system is characterized by its secular approach, traditional pedagogy, and state-run curricula. France’s educational system and pedagogical methods are centralized by the French National Education (Éducation Nationale), which applies national standards to ensure consistency in education up to the university level. The French pedagogical approach is generally academia-focused and especially attentive to the transmission of theoretical knowledge [1]. Students are frequently evaluated by standardized national assessments and in-class quizzes. However, recent amendments have introduced more flexibility to adapt to the individual needs of students and a more holistic approach to supporting children’s cognitive, emotional, and social development [2].

Addressing children’s global development is crucial, especially given recent data showing a concerning prevalence of mental health disorders among children in France. Indeed, about 13% of children aged 6 to 11 years present with a probable mental health disorder, with 12% experiencing anxiety disorders and 5% experiencing mood disorders [3,4]. Conditions such as Attention Deficit Hyperactivity Disorder (ADHD), Obsessive-Compulsive Disorder (OCD), and autism spectrum disorders also affect a significant proportion of children [5,6]. These statistics highlight the need for adaptation and early preventive intervention measures to improve mental health and reduce school dropout rates in children [7].

French primary schools serve children aged 6 to 11 years, spread across five levels (CP to CM2). Teaching methods are based on developmental theories, such as those of Piaget and Vygotsky, which aim to align curricula with children’s cognitive stages [8,9]. Special educational needs, such as those of students with learning difficulties or special needs, are addressed through inclusive arrangements and personalized support [7,10] Despite efforts to make education more inclusive, social, economic, and cultural inequalities persist in the French school system. In response to the challenges exacerbated by the COVID-19 pandemic, the government launched initiatives to strengthen psychological care and mental health procedures in schools, including mobile support teams and mental health awareness programs [11]. Recent revisions in the French National Education system aim to create a school environment that supports not only academic learning but also the emotional well-being of students. Indeed, research suggests that a positive school climate, with caring relationships between teachers, students, and peers, can improve children’s psychological well-being [12]. Conversely, a stressful school environment and conflicting relationships can lead to stress and anxiety [13]. Nonetheless, family support also plays a crucial role: a stable family environment promotes children’s emotional and cognitive development, while family conflicts increase the risk of psychological disorders [14,15]. Therefore, along with family support, studies have highlighted the importance of integrating interventions into educational programs to promote children’s development and resilience [16,17].

Recent literature suggests that school environments have direct effects on students’ learning abilities, with emotions playing a key role in cognitive processes such as attention, working memory, and executive control [18]. Emotional disorders, such as anxiety, can affect these processes, thereby impairing academic performance [19,20]. Effective emotional regulation can reduce negative emotional interference and enhance the use of cognitive resources [21,22]. Emotional consciousness, or the ability to recognize and understand one’s own emotions, is crucial for effective emotion regulation in children. Research shows that children who are more aware of their emotions can better identify and articulate their feelings, which is a key step in managing and regulating their emotional responses [23]. Emotional consciousness allows children to utilize adaptive regulation strategies, such as cognitive reappraisal, which involves reinterpreting a negative emotion in a positive way, thereby reducing its impact [21,22]. Furthermore, fostering emotional awareness in children has been associated with improved social skills, mental health, and academic performance, as it provides a foundation for empathy and better interpersonal relationships [24]. As children’s emotional awareness grows, they become more capable of navigating complex emotional situations, leading to more positive developmental outcomes [25].

Furthermore, France is currently faced with mental health accessibility challenges such as a shortage of mental health professionals, regional disparities, underfunding of services, lack of adequate facilities, and other barriers such as the cost of private care [26]. Therefore, it is important to invest further in preventive and supportive measures in schools. In fact, emphasizing the development of emotional skills of children starting from primary school levels could improve not only the mental health of French children and future adults, but also support students’ cognitive and learning abilities in the long run [2].

Mindfulness-Based Interventions (MBIs), such as Mindfulness-Based Stress Reduction (MBSR) and Mindfulness-Based Cognitive Therapy (MBCT) programs, have been scientifically tested over the past 30 years through rigorous empirical studies. These studies show that MBIs are effective in reducing stress, anxiety, and depressive relapse while improving cognitive functions and quality of life in clinical and general populations, adults, and youth [27,28]. The promotion of mindfulness integration in schools, hospitals, and workplaces has grown exponentially. In an educational context, MBIs aim to improve children’s overall development by enhancing their emotional regulation, cognitive skills, and resilience [29,30,31]. Programs such as “Learning to BREATHE” [32], “MindUp” [33], and “.b” [34] were developed and used internationally in schools to teach mindfulness skills to children. These programs are adapted to the different developmental stages of students and have shown positive results in emotional regulation and cognitive skills, such as attention and concentration. Public initiatives, such as the “Mindful Nation UK” report, have advocated for the integration of mindfulness practices across various institutions. However, debates continue regarding their compatibility with certain secular principles, particularly in France. Indeed, only a very few school mindfulness-based interventions (SMBIs) have been tested in French public schools compared to neighboring francophone countries. In Belgium, SMBIs receive more institutional support and funding, facilitating their systematic integration into schools, while in France, their integration is still in the development phase and often limited to local or experimental initiatives. Therefore, more research is needed to prove the benefits, feasibility, and acceptability of SMBIs in the French public school systems as well as to justify support and funding for wider scale studies. Integrating psychological interventions, such as cognitive-behavioral and mindfulness-based approaches, into school programs can enhance students’ mental health and resilience [35]. Such approaches can improve emotional regulation, cognitive performance, and overall academic success [36]. Thus, the current research evaluates the effectiveness of SMBIs in promoting mental health and well-being in school settings.

## 2. Materials and Methods

### 2.1. Design and Setting

This study was conducted in a school setting in France, involving three public primary schools located in three urban districts of the Ile-de-France region. This exploratory study employed a longitudinal design to evaluate the effectiveness of a mindfulness-based intervention (MBI) on children’s emotional regulation and well-being. The intervention was conducted in a classroom environment, and all procedures were supervised by master’s students in Clinical Psychology and the school’s National Education psychologist.

### 2.2. Participants and Procedure

The sample size was determined using the G Power software (version 3) [http://www.gpower.hhu.de] accessed on 12 October 2019 [37]. For a β power of 0.95, a significance level α of 0.05, and an effect size of 0.25 (medium-large), the minimum required sample size for the experimental group was calculated to be 33 participants. The original sample consisted of 43 children aged 8 to 12 years, from grades CE2 (third grade), CM1 (fourth grade), and CM2 (fifth grade). Three intervention cohorts were recruited over three school years (2019–2020, 2020–2021, and 2022–2023) to ensure statistical power and generalizability. The inclusion criteria were an age range of 8–12 years and proficiency in French to ensure comprehension of the concepts discussed during the sessions. This decision considers the cognitive development level required to benefit from the intervention, suggesting that the “concrete operational stage” between 8 and 12 years is the most suitable age range for the GEPC program [38,39]. Regarding exclusion criteria, children with cognitive impairments or delays incompatible with questionnaire-based evaluation were excluded from the analysis, mainly due to the inadequacy of standardized tools used [40] and to minimize comprehension biases [41] and response biases [42]. Refusal to participate in the group was also an exclusion criterion to preserve the internal and external validity of the results [43], respect participant commitment [44], and uphold research ethics by protecting participants’ rights to decide their engagement in a study [45]. Table 1 summarizes the participants’ characteristics.

**Figure 1:**
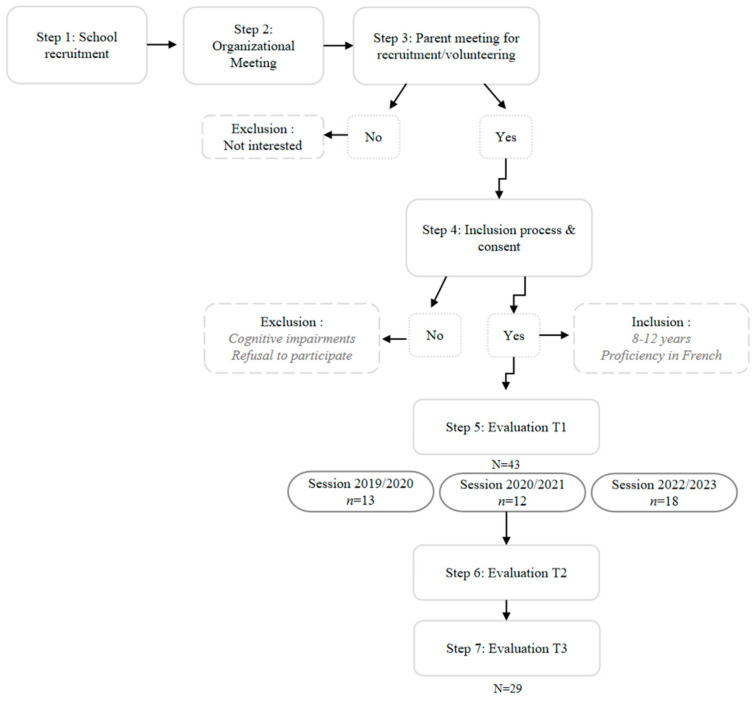
Recruitment process flow chart.

This study was presented to the participants as a pilot study that parents and children could volunteer to participate in. The recruitment process followed five steps (see Figure 1):

Step 1: Contact School Principals and Obtain Permissions: School principals were contacted, and permissions were obtained from the Directors of Education and Childhood.

Step 2: Parent Meeting, obtaining consent, and volunteering: A meeting accessible to all parents and children of the school was held to explain the study and consent forms were distributed. This step was crucial to recruit participants, as parents offered their children’s participation if interested.

Pre-Intervention Evaluation (T1): Baseline data were collected in classrooms supervised by trained psychology students.

Mindfulness-Based Intervention: Conducted over nine weekly sessions, 90 min each.

Post-Intervention Evaluation (T2 and T3): Assessments immediately after the intervention and three months later, following the same procedures.

### 2.3. Data Collection

Firstly, the research protocol was approved by the Ethics and Research Committee of Paris Nanterre University. The administration of the questionnaires co-occurred with a systematic reminder of ethics, anonymity, and the possibility of withdrawal at any time. We conducted three evaluation phases: T1 (before the intervention), T2 (immediately after the intervention), and T3 (three months following the intervention). These assessments took place in school facilities, specifically in a classroom setting after school hours, and were supervised by master’s students in Clinical Psychology and the school’s National Education psychologist. Each session lasted approximately one hour. The parent-reported scale (BRIEF-2) was given to the children to take home and have their parents complete, then returned at the next session. All collected data were anonymized and coded. Data collection took place in a school setting disrupted by the COVID-19 crisis, particularly during the periods of lockdown and class closures in 2020 and 2021. The intervention was offered in two formats: during school hours and after school. This dual approach was necessitated by COVID-19 restrictions, lockdowns, and varying levels of flexibility in school policies, including class schedules and permits. Adapting to what schools were able and willing to accommodate ensured that the intervention could still be implemented effectively. These two formats may have impacted outcomes by influencing attendance, engagement, and consistency, as each format offered different levels of accessibility and integration into students’ routines. These exceptional conditions affected student availability, reducing participation in certain phases of the study, particularly during the follow-up assessments in T3. The final sample consisted of 29 participants, as incomplete protocols and cases with missing data were excluded from the analyses. Due to the lack of complete data for this period, certain statistical analyses, such as Cronbach’s alpha calculations and repeated-measures ANOVAs, could not be performed for this time point. Consequently, these data are marked as NA (not available) in the results table. This limitation is taken into account in the interpretation of the results and will be detailed in the section dedicated to the study’s limitations.

### 2.4. Intervention

The “Emotion Regulation through Mindfulness Practice” (ERMP) in French, “Gestion des Émotions par la Pratique de la Pleine Conscience” (GEPC), program was used in this study [46]. The ERMP program has demonstrated its beneficial effects on francophone youth populations both within and outside school contexts in Belgium. It incorporates psycho-education and practices inspired by MBSR and MBCT techniques to enhance the psychological well-being and cognitive abilities of children and adolescents [47,48]. Additionally, it integrates third-wave Cognitive-Behavioral and Emotional practices, particularly principles from Acceptance and Commitment Therapy (ACT), which emphasize psychological flexibility—enabling participants to adapt effectively to challenging emotions and situations [49]. These practices include fostering acceptance, mindfulness, and commitment to value-based actions that complement the traditional mindfulness techniques.

The program is structured to help children acquire strategies to improve their emotional regulation skills, optimize attentional capacities, and enhance psychological well-being. The intervention involved nine weekly sessions of 90 min, focusing on psycho-education and mindfulness practices suitable for children. Each session focuses on a specific theme, blending theoretical knowledge with experiential practices.

- Psycho-Educational Aspects

The program provides participants with foundational knowledge about emotions, their functions, and the impact of automatic judgments and avoidance behaviors [21]. Psycho-education helps participants recognize the connection between their thoughts, emotions, and bodily sensations, fostering emotional awareness and self-compassion [50].

- Mindfulness Practices

Each session incorporates practical exercises to cultivate mindfulness, attention, and emotional regulation. These include:

Breathing Exercises and Body Scans: Enhancing present-moment awareness and focusing on bodily sensations to improve attention and presence in daily life [47].

Emotion Recognition Practices: Helping participants identify and label emotions [51].

Techniques for Managing Difficult Emotions: Welcoming and accepting negative emotions without judgment [49].

Distancing from Thoughts: Observing thoughts as transient phenomena and developing detachment from their content [48].

Integration Activities: Synthesizing learning and creating a plan for continued practice [52].

The program’s holistic design combines theoretical learning with hands-on practices to build mindfulness skills that promote attention, acceptance, compassion, and emotional regulation, ultimately supporting long-term well-being. However, due to copyright restrictions and the qualifications required to implement this program, the specific details of the session content cannot be shared in this publication.

### 2.5. Outcome Measures

- Executive Functions

The Behavior Rating Inventory of Executive Function, 2nd edition (BRIEF-2) [53] is an 86-item inventory that assesses executive behavior in school and home settings. The parent form is completed by the children’s parents, providing insight into behaviors observed at home. The parent-report version was chosen to minimize the workload for teachers, given that the teacher form (63 items) would impose a substantial burden that could affect result validity [54]. The BRIEF-2 identifies executive dysfunction and its impact on children’s daily lives. It includes scales measuring Inhibition, Flexibility, Emotional Control, Initiation, Working Memory, Planning/Organization, and Organization of Materials, which together provide a Global Executive Composite (GEC) score. The GEC score provides an overall measure of executive functioning by summarizing difficulties in both behavior and cognitive regulation. Higher GEC scores indicate greater executive function difficulties, whereas lower scores suggest better executive functioning. Internal consistency coefficients for the parent form are 0.90 for the Behavioral Regulation Index (BRI), 0.94 for the Metacognition Index (MI), and 0.95 for the GEC.

- Symptomatology: Anxiety & Depression

To assess depression, we used the French version of the Child Depression Inventory (CDI) [55,56]. It consists of 27 items suitable for youth aged 7 to 17 years. Scores are calculated based on age and gender, and a total raw score of 19 or higher indicates a depressive state. The internal consistency of the CDI is high, with a Cronbach’s alpha of 0.70, although its test-retest reliability is low at 0.43 after one month.

To assess anxiety, we used the Revised Children’s Manifest Anxiety Scale (RCMAS) French version [57,58], a self-report questionnaire with 37 items. It has five factors and two global scales and shows good internal consistency (0.87) and test-retest validity (0.67 over six months).

- Emotional Awareness

The Emotion Awareness Questionnaire (EAQ30) [59] is a self-report measure for children and adolescents that assesses awareness of their own and others’ emotions. Suitable for ages 8 to 16, it consists of 30 Likert scale items divided into six sub-scales. The French version shows good psychometric properties, with internal consistency (Cronbach’s alpha of 0.74) and low social desirability bias.

- Mindfulness

The Child and Adolescent Mindfulness Measure (CAMM) [60] is a self-report tool developed to assess mindfulness in children and adolescents. It has 10 items measured on a Likert scale (0 = never true to 4 = always true). Higher scores indicate lower self-acceptance and mindfulness, depending on whether the scores are ascending or descending. The original validation study demonstrated good internal consistency (α = 0.80) and convergent validity.

- School Well-Being

The Be-Scol scale is a French multidimensional measure of school well-being for primary and secondary students [61]. It consists of 28 items across six dimensions and shows good internal consistency and convergent validity. It is divided into 6 dimensions, each consisting of five items: relationships with teachers, school activities, satisfaction with the class, peer relationships, sense of security, and attitude toward evaluations. Regarding psychometric quality, internal consistency (with most alpha coefficients close to 0.70) and test-retest stability are considered satisfactory. Convergent validity is confirmed by the strong link between students’ overall satisfaction and their school well-being. Divergent validity is also confirmed by the weak (or even absent) correlations between the Be-Scol and cognitive performance.

### 2.6. Statistical Analyses

Data were initially imported into Excel (Version 2408) and analyzed using JAMOVI (version 2.5.4). The entire sample was grouped for analyses. We first conducted descriptive analyses to better characterize the sample of children included in the present study. Reliability analyses were conducted using Cronbach’s alpha, particularly regarding the normal distribution of the variables. We conducted parametric repeated-measures ANOVAs to examine the potential impact of the mindfulness intervention on several variables of interest (anxiety, depression, mindfulness, emotional awareness, executive functions, emotion regulation, and school well-being), with session as the within-subjects factor in 3 modalities (T1, T2, and T3, for pre-intervention, immediately post-intervention, and 3 months post-intervention, respectively). The Greenhouse-Geisser correction was applied to account for repeated measures. Tukey’s post-hoc tests were used to perform pairwise comparisons to more precisely examine any observed differences between the sessions (T1, T2, and T3) on the variables of interest. Partial eta squared was used to measure the effect size in the context of ANOVAs. When the amount of data was insufficient, particularly in T3, we only considered sessions T1 and T2, leading us to perform paired-sample *t*-tests. In such cases, Cohen’s d was used to calculate the effect size.

## 3. Results

### 3.1. Descriptive Statistics

Before analyzing the effects of the intervention, descriptive statistics were computed for all dependent variables at each time point (e.g., pre-test (T1), post-test (T2). Follow-up scores (T3) were based on a reduced subsample (n = 14) and did not differ significantly from the post-intervention scores.

The intervention was successfully implemented as planned, and participants attended almost all sessions. We counted for six recorded absences, and the overall absence rate during the intervention was approximately 1.55%. The final sample consisted of 29 participants, as incomplete protocols and cases with missing data were excluded from the analyses. Additionally, attrition occurred due to the COVID-19 lockdowns affecting data collection for the 2020 and 2021 groups, and one child from the 2023 group left the intervention midway because they changed schools.

Table 2 presents the descriptive statistics of all outcome measures in the pre-, post-intervention, and follow-ups.

### 3.2. Reliability Analysis

The reliability analysis for the measures used in the study showed acceptable to good internal consistency across sessions. Cronbach’s alpha values for the CDI ranged from 0.78 at T1 to 0.91 at T3, indicating improved reliability over time. For the RCMAS, values were consistent across sessions, ranging from 0.78 at T1 and T3 to 0.81 at T2. The CAMM showed slight improvements, with alpha values increasing from 0.67 at T1 to 0.71 at T2 and T3. The EAQ-30 demonstrated variability, with alpha values improving from 0.70 at T1 to 0.84 at T2 but decreasing to 0.67 at T3. For the BE-SCOL, reliability was highest at T1 (0.93) and T3 (0.83) but dropped to 0.60 at T2. Parent-reported measures of executive functions (BRIEF) showed strong internal consistency, with Cronbach’s alpha values of 0.96 (mothers) and 0.91 (fathers) at T1, and 0.97 (mothers) and 0.92 (fathers) at T2. T3 data for the BRIEF were not available due to missing data.

### 3.3. Efficacy of the School Mindfulness Intervention

The findings of the repeated-measures ANOVAs are presented in Table 3. Results of *t*-test measures for executive function outcomes are presented in Table 4.

- Anxiety

A repeated-measures analysis of variance (ANOVA) was conducted to examine the effect of sessions on the total RCMAS scores measured across the three time points (T1, T2, and T3). Mauchly’s test of sphericity did not indicate a significant violation (W = 0.742, *p* = 0.205), but Greenhouse-Geisser (ε = 0.821) and Huynh-Feldt (ε = 0.876) corrections were applied as appropriate to account for any potential sphericity issues.

The analysis did not reveal a significant main effect of session on the total RCMAS scores (F(1.64, 43.93) = 2.12, *p* = 0.142, η^2^_p_ = 0.09). Post-hoc comparisons also showed no statistically significant differences between T1 and T2, T1 and T3, or T2 and T3 (all *p*-values > 0.05). The estimated marginal means for the total RCMAS scores were 13.1 (SD = 1.71) at T1, 12.8 (SD = 1.43) at T2, and 11.2 (SD = 1.25) at T3. While there was a slight reduction in scores over time, the changes were not statistically significant. This finding is consistent with the sub-scale scores (Physiological Anxiety, Worry/Over-sensitivity, and Social Concerns/Concentration), which also showed no significant changes across sessions. These results suggest that the intervention did not produce measurable reductions in anxiety, as assessed by the RCMAS.

- Depression

Repeated-measures analysis of variance (ANOVA) was conducted to examine the effect of session on total CDI (Children’s Depression Inventory) scores measured across three time points (T1, T2, and T3). The analysis revealed that the main effect of session was not significant, F(1.73, 20.81) = 0.202, *p* = 0.788, η^2^_p_ = 0.017, with a Greenhouse-Geisser correction applied. This suggests that the total CDI scores did not significantly vary across the three sessions. Mauchly’s test of sphericity did not indicate a significant violation (W = 0.847, *p* = 0.401); therefore, no major corrections were necessary. Greenhouse-Geisser (ε = 0.867) and Huynh-Feldt (ε = 1.00) corrections were applied for robustness. Post-hoc comparisons between sessions revealed no significant differences. The difference between T1 and T2 was −0.846, t(12) = −0.470, *p* = 0.886, and the difference between T1 and T3 was 0.615, t(12) = 0.243, *p* = 0.968. Similarly, there was no significant difference between T2 and T3, with a mean difference of 1.462, t(12) = 0.581, *p* = 0.833. The estimated marginal means for total CDI scores were 13.0 (1.76) at T1, 13.8 (2.56) at T2, and 12.4 (2.72) at T3. These results suggest that the total CDI scores remained stable across the three sessions, with no significant variation observed over time.

- Mindfulness

Repeated-measures analysis of variance (ANOVA) was conducted to examine the effect of session on total CAMM (Children and Adolescent Mindfulness Measure) scores measured across three time points (T1, T2, and T3). The analysis revealed that the main effect of session was not significant, F(1.99, 23.86) = 3.07, *p* = 0.065, η^2^_p_ = 0.204, with a Greenhouse-Geisser correction applied. This suggests that total CDI scores did not significantly vary across the three sessions. Mauchly’s test of sphericity did not indicate a significant violation (W = 0.994, *p* = 0.969); therefore, no major corrections were necessary. Greenhouse-Geisser (ε = 0.994) and Huynh-Feldt (ε = 1.00) corrections were applied for robustness. Post-hoc comparisons between sessions revealed no significant differences. The difference between T1 and T2 was 1.46, t(12) = 0.591, *p* = 0.828, and that between T1 and T3 was 5.69, t(12) = 2.435, *p* = 0.075. Similarly, there was no significant difference between T2 and T3, with a mean difference of 4.23, t(12) = 1.806, *p* = 0.209). The estimated marginal means for total CAMM scores were 19.6 (1.80) at T1, 18.2 (1.20) at T2, and 13.9 (1.90) at T3. These results suggest that the total CAMM scores remained stable across the three sessions, with no significant variation observed over time. However, the results showed a trend toward significance for session effects (*p* = 0.065) on Mindfulness improvement [62].

- Emotional Awareness

Repeated-measures analysis of variance (ANOVA) was conducted to examine the effect of session on the total EAQ-30 (Emotion Awareness Questionnaire) scores measured across three time points (T1, T2, and T3). The analysis revealed that the main effect of session was not significant, F(1.53, 18.31) = 0.597, *p* = 0.518, η^2^_p_ = 0.047, with a Greenhouse-Geisser correction applied. This suggests that the total emotional awareness scores did not significantly vary across the three sessions. Mauchly’s test of sphericity did not indicate a significant violation (W = 0.689, *p* = 0.129); therefore, no major corrections were necessary. Greenhouse-Geisser (ε = 0.763) and Huynh-Feldt (ε = 0.851) corrections were applied for robustness. Post-hoc comparisons between sessions revealed no significant differences. The difference between T1 and T2 was −1.46, t(12) = −0.832, *p* = 0.691, and the difference between T1 and T3 was 1.23, t(12) = 0.411, *p* = 0.912. Similarly, there was no significant difference between T2 and T3, with a mean difference of 2.69, t(12) = 1.079, *p* = 0.544). The estimated marginal means for total EAQ-30 scores were 60.5 (2.05) at T1, 62.0 (1.82) at T2, and 59.3 (1.99) at T3. These results suggest that total Emotion Awareness scores remained stable across the three sessions, with no significant variation observed over time.

- School Well-Being
  - Total School Well-Being

Repeated-measures analysis of variance (ANOVA) was conducted to examine the effect of session on total Be-Scol (School well-being) scores measured across three time points (T1, T2, and T3). The analysis revealed that the main effect of session was not significant, F(1.46, 17.49) = 0.244, *p* = 0.717, η^2^_p_ = 0.020, with a Greenhouse-Geisser correction applied. This suggests that total School well-being scores did not significantly vary across the three sessions. Mauchly’s test of sphericity did not indicate a significant violation (W = 0.628, *p* = 0.077); therefore, no major corrections were necessary. Greenhouse-Geisser (ε = 0.729) and Huynh-Feldt (ε = 0.804) corrections were applied for robustness. Post-hoc comparisons between sessions revealed no significant differences. The difference between T1 and T2 was −0.231, t(12) = −0.0951, *p* = 0.995), and the difference between T1 and T3 was 2.077 (t(12) = 0.4664, *p* = 0.888). Similarly, there was no significant difference between T2 and T3, with a mean difference of 2.308, t(12) = 0.6150, *p* = 0.815. The estimated marginal means for total Be-Scol scores were 84.6 (3.40) at T1, 84.8 (3.22) at T2, and 82.5 (3.51) at T3. These results suggest that total School well-being scores remained stable across the three sessions, with no significant variation observed over time.

- Executive Functions
  - BRIEF-2: Global Executive Composite (GEC) scores reported by mothers

Due to missing data, a paired samples *t*-test was conducted to compare Global Executive Composite (GEC) reported by mothers between T1 and T2 in a sample of nine participants. The results showed no statistically significant difference between T1 (M = 152, SD = 33.2) and T2 (M = 143, SD = 30.7), t(8) = 1.04, *p* = 0.165, indicating that the observed reduction in scores from T1 to T2 was not significant. The effect size, measured by Cohen’s d, was 0.346, suggesting a small effect. Tests of normality (Shapiro-Wilk, Kolmogorov-Smirnov, and Anderson-Darling tests) confirmed that the data were normally distributed (all *p* > 0.05). Overall, although there was a reduction in GEC scores from T1 to T2, the change was not statistically significant, and the data violated the assumptions of normality.

 
  - BRIEF-2: Global Executive Composite (GEC) scores reported by father

Again, due to missing data, a paired samples *t*-test was conducted to compare Global Executive Composite (GEC) reported by fathers between T1 and T2 in a sample of 11 participants. The results showed no statistically significant difference between T1 (M = 140, SD = 17.4) and T2 (M = 116, SD = 59.6), t(10) = 1.27, *p* = 0.116, indicating that the observed decrease in scores from T1 to T2 was not statistically significant. The effect size, measured by Cohen’s d, was 0.384, suggesting a small-to-medium effect. However, tests of normality (Shapiro-Wilk and Anderson-Darling) revealed a significant deviation from normality in both T1 and T2 scores (*p* < 0.001), which should be considered when interpreting the results. Overall, although there was a reduction in GEC scores from T1 to T2, the change was not statistically significant, and the data violated assumptions of normality.

## 4. Discussion

- Aims and Hypotheses

This study explored the short- and long-term efficacy of a school mindfulness intervention, specifically the GEPC program, in French public primary schools. The primary hypothesis was that the intervention would lead to significant improvements in anxiety, depression, emotional awareness, mindfulness, executive functioning, and school well-being. While some numerical trends aligned with the hypotheses, no statistically significant improvements were observed across the investigated dimensions. These findings provide insight into the limitations of the intervention design and the measures used to assess its effectiveness, emphasizing the importance of adapting both the program structure and evaluation tools to better capture potential benefits in future studies.

Despite the lack of significant effects, the slight numerical reductions in anxiety scores and stable depression levels suggest potential avenues for exploring how mindfulness interventions can maintain well-being in specific contexts. These findings align with prior literature highlighting the variability in SMBIs’ effectiveness depending on population, intervention structure, and implementation conditions [30,63].

- Anxiety and Depression

The intervention did not significantly reduce anxiety or depression symptoms. This could be attributed to the relatively short duration of the program or its scope, which may not have been sufficient to target specific anxiety subtypes or depressive symptoms. Additionally, individual variability in responsiveness to mindfulness practices and measurement sensitivity of tools like the CDI likely influenced these results [64,65].

- Mindfulness

The observed slight improvements in mindfulness (CAMM) were not statistically significant. Factors such as developmental limitations in children’s understanding of mindfulness concepts and the unidimensional nature of the CAMM may have contributed to these results. Recent literature emphasizes the need for more developmentally appropriate, multidimensional tools to assess mindfulness in young populations [66,67].

- Emotional Awareness, Executive Functioning, and School Well-being

Contrary to expectations, the intervention did not significantly improve emotional awareness, executive functioning, or school well-being. These findings contrast with those of previous studies reporting the positive effects of SMBIs on these outcomes [33,68]. Parent-reported measures and contextual factors, such as the school environment, likely influenced these outcomes, suggesting the need for more targeted strategies or tailored interventions.

- Limitations and future directions

Several limitations of this study should be considered when interpreting these findings. First, the lack of meaningful differences across sessions can be explained by the small sample size, which may have reduced the power to detect significant changes in some outcomes. Larger studies might reveal more subtle differences that these analyses could not detect. For instance, the observed slight increase from T1 to T2 (from 13.0 to 13.8) in depression scores and the subsequent decrease to 12.4 at T3, while non-significant in this small sample, could become significant with more participants, especially if these trends reflect true underlying changes in depression symptoms. This highlights the need for future studies with larger sample sizes to more effectively evaluate the potential impact of the intervention on anxiety outcomes. Given these results, future research should consider extending the intervention duration, incorporating targeted strategies for specific types of anxiety, and introducing follow-up sessions to sustain potential gains. Larger sample sizes and longitudinal follow-up (e.g., at 6 and 12 months) could also help determine the long-term trajectory of anxiety-related changes and offer deeper insights into the mechanisms of SMBIs in reducing childhood anxiety.

Second, future studies should aim to address the lack of randomization and the potential influence of contextual differences between schools. In this study, the sample consisted of three groups from different schools, and inclusion was based on parental interest rather than random selection. This introduces the risk of selection bias and limits the generalizability of the findings. Furthermore, differences between schools, such as variations in school resources, teaching quality, or the socioeconomic status of the neighborhoods, which were not measured in this study, may have confounded the results, making it difficult to attribute observed changes solely to the SMBI. Indeed, the sample lacked detailed documentation of participant diversity, such as socioeconomic, ethnic, or cultural backgrounds. Including such information in future studies would provide important insights into how diverse populations respond to SMBIs and enhance the generalizability of the findings. Recruiting more diverse samples and employing randomization methods, such as cluster randomization, could mitigate these biases and offer a clearer understanding of the intervention’s effectiveness across different demographic and contextual settings.

The absence of a control group in this study represents a key limitation, as it restricts the ability to attribute observed changes exclusively to the intervention. While the initial study design included a wait-list control group composed of children attending the study hall, this group was lost during the COVID-19 lockdown, as study-hall sessions were no longer offered after the first lockdown. Additionally, the practical constraints imposed by school policies on the 2023 group limited our ability to include a control group. To address this limitation in future research, employing a cluster randomization design could be particularly beneficial. In this approach, entire schools or classrooms, rather than individual participants, are randomly assigned to either the intervention or control condition. This method minimizes contamination between groups, as students in the same school or classroom share similar environments and are less likely to influence each other’s outcomes. Additionally, cluster randomization aligns well with the practical constraints of implementing school-based interventions and ensures ecological validity by maintaining a natural classroom setting [69].

These findings should be interpreted with caution. Indeed, data were missing due to COVID-19 disruptions, participant withdrawal or non-response, and specific school functioning (school and class closures), which are common challenges in extraordinary contexts such as pandemics [70], but also common in longitudinal research [71]. The missing data may have introduced a potential bias, affecting the generalizability of the results. Future research should analyze the extent and pattern of missing data to explore whether they could influence outcomes. This study’s primary focus was on exploring overall trends rather than modeling complex hierarchical effects, making simpler statistical methods, such as repeated-measures ANOVA, more appropriate for this context. However, longitudinal research where attrition risk is high could benefit from applying Multilevel Modeling (MLM) statistical analyses, which offer significant advantages over traditional approaches. MLM is well suited for handling incomplete data, as it allows for the inclusion of all available data points without requiring listwise deletion, thereby maximizing the use of the sample [72]. This is particularly beneficial in studies where participants may drop out or fail to complete all time points, a common challenge in longitudinal mindfulness-based intervention (MBI) research. Furthermore, MLM can model individual trajectories of change, capturing the variability between participants in response to the intervention, which is critical for understanding nuanced patterns in psychological outcomes [73]. Applying MLM in future studies could yield deeper insights into the effectiveness of interventions despite attrition, ensuring that the results remain reliable and representative. As pointed out by [74], “MLM provides a more comprehensive framework for studying within- and between-person effects,” making it particularly advantageous for capturing dynamic changes in outcomes such as anxiety and emotional regulation over time. Leveraging these methods can enhance the rigor and interpretability of longitudinal findings while addressing the common pitfalls of participant dropout and incomplete data.

In addition, the SMBI’s length and content may not have been sufficient to produce more profound changes. We suggest a need for longer programs (12 sessions) or booster sessions post-program, which could be beneficial in reaching the desired effects and maintaining them through time. However, shorter and more focused interventions targeting specific issues, such as anxiety or depression, could improve their effectiveness. For instance, primary prevention programs like “The Penn Resiliency Program,” which is aimed at promoting overall well-being and preventing depression in the general population, have demonstrated success in reducing depressive symptoms and improving resilience [75]. While these interventions do not include mindfulness-based components, other approaches, such as the weekly practice of recalling and elaborating episodes of gratitude, self-affirmation, goal setting, or meaningful things, have been shown to improve well-being in university students by fostering positive emotions, gratitude, and a sense of purpose [76]. These practices, such as reflective practices and positive elaboration, can be integrated into school mindfulness-based interventions to enhance their impact on both mental health and resilience.

Finally, reliance on self-report measures, particularly with younger children, may have introduced bias or inaccuracies, as children may struggle to accurately assess and report their internal states [77]. Exploring innovative methods, such as observational measures or state-based tools, could enhance future research by capturing a more comprehensive picture of mindfulness in children.

## 5. Conclusions

This study adds valuable insight and perspectives into mindfulness research, particularly regarding school MBI’s in a French public primary school context. However, the intervention failed to produce substantial changes in mindfulness, anxiety, depression, emotional awareness, executive functions, and school well-being. These results suggest that further investigation and refinement of SMBIs are needed to maximize their effectiveness and adaptation to the French school system.

## Figures and Tables

**Table 1:** Participants’ Demographic Characteristics.

Variable	N	Mean (M)	(SD)	Median	Min.	Max.	Frequency (n)	% of Total	Cumulative %
Age (months)	43	116.00	9.06	115	99	132			
**Gender**									
Boys							20	46.5%	46.5%
Girls							23	53.5%	100.0%
**Class**									
CE2							15	34.9%	34.9%
CM1							26	60.5%	95.3%
CM2							2	4.7%	100.0%
**Format**									
After-class							25	58.1%	58.1%
In-class							18	41.9%	100.0%
**Familial Status**									
Married							26	60.5%	60.5%
Divorced							9	20.9%	81.4%
Separated							4	9.3%	90.7%
Cohabitation							4	9.3%	100.0%
**Pandemic**									
During							25	58.1%	58.1%
Post							18	41.9%	100.0%

Note: CE2 (aged 8 to 9), CM1 (aged 9 to 10), and CM2 (aged 10 to 11).

**Table 2:** Descriptive statistics of participant outcome marginal means.

	Pre-Intervention (T1)	Post-Intervention (T2)
Outcomes	M (SD)	M (SD)
N	29	27
**Anxiety**		
Physiological Anxiety	4.38 (0.63)	4.38 (0.66)
Worry/Over-sensitivity	5.85 (0.52)	5.92 (0.67)
Social Concerns/concentration	2.85 (0.60)	2.46 (0.50)
Total	**13.1** **(1.71)**	**12.8** **(1.43)**
**Depression**		
Negative Mood	3.46 (0.72)	3.69 (0.84)
Interpersonal Problems	1.23 (0.38)	1.23 (0.44)
Ineffectiveness	2.38 (0.33)	2.46 (0.46)
Anhedonia	4.31 (0.89)	4.62 (0.87)
Negative self-esteem	1.62 (0.43)	1.85 (0.66)
Total	**13 (1.76)**	**13.8 (2.56)**
**Mindfulness**		
Mindfulness	**19.6 (1.80)**	**18.2 (1.20)**
**Emotional Awareness**		
Emotional Awareness	**60.5 (2.05)**	**62.0 (1.82)**
**School Well-being**		
Teacher relationship	16.1 (0.79)	15.8 (0.97)
School activities	12.8 (1.13)	12.6 (0.90)
Class satisfaction	16.8 (0.82)	16.2 (1.07)
Peer relationships	15.0 (0.81)	14.8 (0.78)
Sense of security	12.0 (1.12)	12.9 (0.84)
Evaluations	11.8 (0.98)	12.5 (0.84)
Total	**84.6 (3.40)**	**84.8 (3.22)**
**Executive functions**		
Mother-reported (GEC)	**152 (11.1)**	**143 (10.2)**

Note: follow-up scores at T3 do not differ significantly from post-intervention scores.

**Table 3:** Results of Repeated-Measures ANOVAs at each experimental session.

Outcomes		Pre–Post (T1–T2)			Pre-Follow Up (T1–T3)			Post-Follow Up (T2–T3)		
	η^2^_p_	M Df	t	p_tkey_	M Df	t	p_tkey_	M Df	t	p_tkey_
Anxiety (RCMAS)										
Physiological Anxiety	0.09	0.00	0.00	1.00	0.69	1.29	0.43	0.69	1.17	0.49
Worry/ Over-sensitivity	0.05	−0.08	−0.16	0.97	0.69	0.86	0.67	0.77	0.81	0.70
Social Concerns/ concentration	0.06	0.38	1.24	0.46	0.46	1.07	0.56	0.08	0.16	0.97
Total RCMAS	0.09	0.31	0.44	0.898	1.85	1.40	0.37	1.54	0.97	0.61
Depression (CDI)										
Negative Mood	0.02	−0.23	−0.36	0.93	0.46	0.55	0.85	0.69	0.72	0.76
Interpersonal Problems	0.04	−2.22e	−6.93e	1.00	0.31	0.81	0.71	0.31	0.77	0.73
Ineffectiveness	0.01	−0.08	−0.18	0.98	−0.15	0.31	0.95	−0.15	0.31	0.95
Anhedonia	0.02	−0.31	0.44	0.90	0.23	0.30	0.95	0.54	0.76	0.74
Negative self-esteem	0.01	−0.23	−0.36	0.93	−0.23	−0.26	0.96	4.44 × 10^−16^	5.15 × 10^−16^	1.00
Total CDI	0.02	−0.85	−0.48	0.87	0.62	0.24	0.97	1.462	0.58	0.83
Mindfulness (CAMM)										
Total CAMM	0.20	1.46	0.60	0.83	5.69	2.44	0.08	4.23	1.81	0.21
Emotion Awareness (EAQ-30)										
Total EAQ-30	0.05	−1.46	−0.83	0.70	1.23	0.41	0.91	2.69	1.08	0.54
School Well-being (Be-Scol)										
Teacher relationship	0.12	0.23	0.25	0.97	1.70	1.69	0.25	1.46	1.33	0.40
School activities	0.02	0.231	0.25	0.97	−0.54	−0.38	0.93	−0.77	−0.71	0.76
Class satisfaction	0.04	0.69	0.81	0.71	0.70	0.82	0.70	3.55 × 10^−15^	4.48 × 10^−15^	1.00
Peer relationships	0.01	0.15	0.22	0.97	0.54	0.48	0.89	0.39	0.38	0.93
Sense of security	0.10	−0.92	−0.86	0.68	0.70	0.65	0.80	1.62	2.19	0.11
Evaluations	0.03	−0.62	−0.53	0.86	−1.00	−0.99	0.60	−0.39	−0.34	0.94
Total Be-Scol	0.20	−0.231	−0.10	1.00	2.08	0.47	0.90	2.31	0.62	0.82

Note: η^2^_p_; Parietal eta squared; M Df, Mean Difference; t, t value, p_tkey_, Tukey’s Honestly Significant Difference.

**Table 4:** Results of *t*-test measures for executive function outcomes.

Outcomes	Pre–Post (T1–T2)		
	t	*p*	Cohen’s d
BRIEF-2 reported by mothers			
Total GEC	1.04	0.17	0.35
BRIEF-2 reported by fathers			
Total GEC	1.27	0.12	0.38

Note: Due to insufficient data in T3, paired-sample *t*-tests were performed between sessions T1 and T2; t, t value; *p*, *p* value; Cohen’s d, Effect Size.

## Data Availability

The data presented in this study are available upon request from the corresponding author due to privacy and ethical restrictions.
